# Genome-Wide Identification of the CYP716 Gene Family in *Platycodon grandiflorus* (Jacq.) A. DC. and Its Role in the Regulation of Triterpenoid Saponin Biosynthesis

**DOI:** 10.3390/plants13141946

**Published:** 2024-07-16

**Authors:** Wuhua Zhang, Javed Iqbal, Zhihui Hou, Yingdong Fan, Jie Dong, Chengzhi Liu, Tao Yang, Daidi Che, Jinzhu Zhang, Dawei Xin

**Affiliations:** 1 College of Horticulture and Landscape Architecture, Northeast Agricultural University, Harbin 150030, China; 15346549367@163.com (W.Z.); iqbal468@aup.edu.pk (J.I.); 18634445324@163.com (Z.H.); fanying_dong@163.com (Y.F.); jiedong1521@163.com (J.D.); liuchengzhi1103@163.com (C.L.); yangtao@neau.edu.cn (T.Y.); daidiche@neau.edu.cn (D.C.); 2 Key Laboratory of Cold Region Landscape Plants and Applications, Harbin 150030, China; 3 Key Laboratory of Soybean Biology in Chinese Ministry of Education, College of Agriculture, Northeast Agricultural University, Harbin 150030, China

**Keywords:** *P. grandiflorus*, triterpenoid saponins, gene family analysis, CYP716, oleanolic acid

## Abstract

The main type of saponins occurring in the root of *Platycodon grandiflorus* (Jacq.) A. DC. are oleanolic acid glycosides. The *CYP716* gene family plays a major role in catalyzing the conversion of β-amyrin into oleanolic acid. However, studies on the *CYP716* genes in *P. grandiflorus* are limited, and its evolutionary history remains poorly understood. In this study, 22 *PgCYP716* genes were identified, distributed among seven subfamilies. *Cis*-acting elements of the PgCYP716 promoters were mainly involved in plant hormone regulation and responses to abiotic stresses. *PgCYP716A264*, *PgCYP716A391*, *PgCYP716A291*, and *PgCYP716BWv3* genes were upregulated in the root and during saponin accumulation, as shown by RNA-seq analysis, suggesting that these four genes play an important role in saponin synthesis. The results of subcellular localization indicated that these four genes encoded membrane proteins. Furthermore, the catalytic activity of these four genes was proved in the yeast, which catalyzed the conversion of β-amyrin into oleanolic acid. We found that the content of β-amyrin, platycodin D, platycoside E, platycodin D3, and total saponins increased significantly when either of the four genes was over expressed in the transgenic hair root. In addition, the expression of *PgSS*, *PgGPPS2*, *PgHMGS*, and *PgSE* was also upregulated while these four genes were overexpressed. These data support that these four PgCYP716 enzymes oxidize β-amyrin to produce oleanolic acid, ultimately promoting saponin accumulation by activating the expression of upstream pathway genes. Our results enhanced the understanding of the functional variation among the *PgCYP716* gene family involved in triterpenoid biosynthesis and provided a theoretical foundation for improving saponin content and enriching the saponin biosynthetic pathway in *P. grandiflorus*.

## 1. Introduction

*Platycodon grandiflorus* (Jacq.) A. DC. is a perennial herbaceous plant of the Campanulaceae family. It is an economically important plant with ornamental, medicinal, and edible values [1]. It is a traditional herb in Chinese, Korean, and Japanese medicine. Its roots are rich in various bioactive compounds such as saponins, polysaccharides, and flavonoids. Among them, saponins, the primary bioactive components of *P. grandiflorus*, exhibit antitumor and hepatoprotective properties [2,3,4,5]. In recent years, the unique role of traditional Chinese medicines, including *P. grandiflorus*, in treating COVID-19 has gradually led to their increased demand and status [6].

Triterpenoid saponins are glycosides consisting of sapogenins combined with sugars. Most triterpenoids discovered so far are tetracyclic and pentacyclic triterpenoids, with the most common structure being the dammarane-type and oleanane-type [7]. Tetracyclic triterpenes are widely distributed in nature; ginsenosides and gibberellin are tetracyclic triterpenoids [8]. Pentacyclic saponins are more commonly observed in Chinese herbs such as *Glycyrrhiza uralensis*, *Bupleurum chinense*, and *Polygala tenuifolia* [9]. The saponins are mainly oleanane-type pentacyclic triterpenoids in *P. grandiflorus*, of which the main sapogenins is oleanolic acid. Plants mainly derive triterpenoids from the cytosolic mevalonic acid pathway (MVA), with a smaller contribution from the plastidial methylerythritol phosphate pathway (MEP) [10]. Farnesyl pyrophosphate (FPP) is derived from the precursor isopentenyl diphosphate (IPP) through a series of enzymatic reactions. With the assistance of key enzymes such as squalene epoxidase (SE) and farnesyl-diphosphate synthase (FPPS), Farnesyl-PP is transformed into 2,3-oxidosqualene. Subsequently, 2,3-oxidosqualene is cyclized by 2,3-oxidosqualene cyclase (OSC) to yield β-amyrin [11,12,13]. Further, β-amyrin is modified by cytochrome P450 to form oleanolic acid [14].

Cytochrome P450 is a class of heme-based B-type cytochrome superfamily protein that extensively participates in the biosynthesis of specialized metabolites (e.g., terpenes, plant hormones, and alkaloids) in plants [15]. The main enzymes involved in catalyzing the synthesis of oleanolic acid from β-amyrin are from the CYP716 family of the cytochrome P450 superfamily [16]. MtCYP716A12 is the first identified enzyme involved in the synthesis of pentacyclic triterpene saponins, which catalyzes the C-28 oxidation of β-amyrin into oleanolic acid [17]. With the development of sequencing technology, numerous CYP716 enzymes involved in catalyzing oleanolic acid formation have been identified and validated [18,19,20]. In *Kalopanax septemlobus* and *Chenopodium quinoa*, KsCYP716A94, CqCYP716A78, and CqCYP716A79 catalyze the C-28 oxidation of β-amyrin into oleanolic acid [21,22]. Meanwhile, five CYP716 enzymes (including PgCYP716A140, PgCYP716A141, PgCYP716D58, PgCYP716S7, and PgCYP716S6) were identified and validated in *P. grandiflorus* that oxidize β-amyrin at the C-28 position to produce oleanolic acid [18,23]. In addition to oxidizing β-amyrin at the C-28 position to produce oleanolic acid, some CYP716s catalyze triterpene oxidation at different positions, including C-6, C-2α, and C-12 [24,25,26]. For example, PgCYP716A47 catalyzes the C-12 hydroxylation of dammarenediol-II to form protopanaxadiol [27]. Subsequently, under the catalysis of CYP716A52v2, protopanaxadiol is oxidized to produce the downstream product, protopanaxatriol [28].

*P. grandiflorus* is an important traditional Chinese medicinal plant with a wide cultivation area [29]. Oleanolic acid, a pentacyclic oleanane-type triterpene, is the main active ingredient in *P. grandiflorus* [30]. The CPY716 family plays a major role in catalyzing the oxidation of β-amyrin into oleanolic acid. However, a systematic understanding of the *CYP716* gene family and its evolutionary history is lacking. In this study, a total of 22 *PgCYP716* genes were identified, distributed among seven subfamilies. Analysis of the *cis*-acting elements of *PgCYP716* genes revealed that they were mainly involved in plant hormone regulation and responses to abiotic stresses. The *PgCYP716A264*, *PgCYP716A391*, *PgCYP716A291*, and *PgCYP716BWv3* genes were cloned for subsequent experiments. The results of subcellular localization, yeast heterologous expression, and transgenic hairy root transformation indicated that all four enzymes were membrane proteins and oxidized β-amyrin to produce oleanolic acid. This study enhanced the understanding of the saponin synthesis pathway, expanding the gene pool for saponin biosynthesis and accelerating molecular breeding. Moreover, this study provided genetic resources for the cultivation of high-efficiency and high-quality varieties of *P. grandiflorus*.

## 2. Results

### 2.1. Identification of PgCYP716 Gene Family

**Figure 1:**
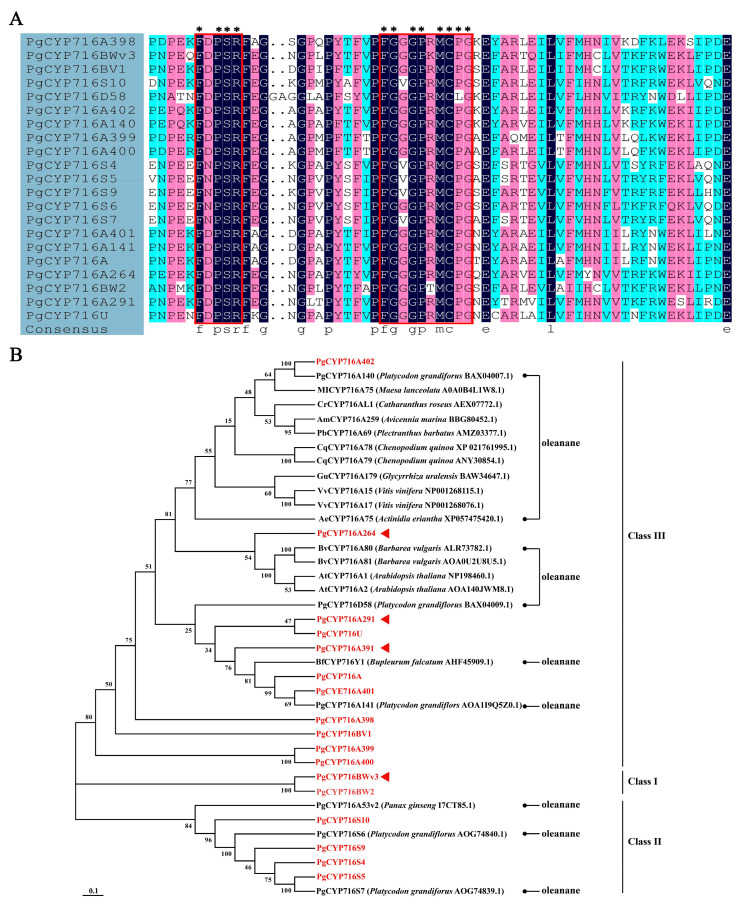
Identification of *PgCYP716s* in *P. grandiflorus*. (**A**) Multiple sequence alignment of the CYP450 domain of the PgCYP716 gene family. The asterisk denoted identical amino acids. (**B**) Phylogenetic tree of CYP716 proteins. The evolutionary tree was constructed based on the complete amino acid sequences of CYP716 proteins via MEGA 11 with the Maximum likelihood method. Bootstrap = 1000. CYP716s identified in this research are highlighted in red, while the filled arrowheads signify those CYP716s that have been functionally characterized in this study.

Following the screening of the *P. grandiflorus* genome, 22 PgCYP716 genes were identified and their amino acid sequences were subsequently forwarded to the CYP450 Family Committee for nomenclature. The P450 family contained the complete P450 helix K region sequence FXXXR and the heme binding motif FXXGXXXCXG/A [31]. Multiple sequence alignment results indicated that all 22 genes contained the complete P450 helix K region sequence and the heme binding motif (Figure 1A). These 22 genes belonged to seven subfamilies, with the CYP716A subfamily containing 11 genes and the CYP716S subfamily containing six genes. The remaining subfamilies were CYP716BV, CYP716BW, CYP716D, CYP716U, and CYP716BWv, each containing only one gene. The length of the coding sequence of the 22 *PgCYP716* genes ranged from 288 to 1656 bp, with *PgCYP716BW2* being the longest, encoding 552 amino acids with a MW of 62,701 Da. PgCYP716A was the shortest, encoding 96 amino acids with a MW of 10,995.45 Da. The pI of the proteins within the PgCYP716s exhibited a wide range, with PgCYP716S5 having the lowest pI at 5.88, while PgCYP716A140 displayed the highest pI at 9.17. This variation in pI values among different members of the PgCYP716s highlighted the diverse biochemical properties and potential functional roles of these proteins (Supplementary Table S1).

### 2.2. Evolutionary Analysis of the PgCYP716 Proteins in Different Species

In order to enhance our comprehension of the evolutionary connections within the PgCYP716 gene family, we performed a multiple sequence alignment of the 22 identified PgCYP716 amino acid sequences from *P. grandiflorus*. Further, 16 PgCYP716 amino acid sequences from 11 other plants, which oxidized β-amyrin to produce oleanolic acid, were used to construct an evolutionary tree. CYP716 proteins could be categorized into three major classes. Class I mainly included the PgCYP71BW and PgCYP716BWv subfamilies, and Class II mainly included the CYP716S subfamily from *P. grandiflorus* and PgCYP716A53v2 from *P. ginseng*. This suggested that the PgCYP716S subfamily may have the same function as the PgCYP716A53v2 of *Panax ginseng* in catalyzing the conversion of protopanaxatriol into protopanaxadiol. The remaining PgCYP716A, PgCYP716D, PgCYP716U, and PgCYP716BV subfamilies were grouped together with the CYP716A family of other species. This indicated that these four subfamilies, along with the majority of the CYP716A family, had the function of synthesizing oleanolic acid (Figure 1B). This analysis provided valuable insights into the evolutionary relationships among the CYP716 gene family in *P. grandiflorus*, establishing a basis for future explorations into the functional properties of this gene family.

### 2.3. Analysis of Conserved Motif and Structure of the PgCYP716 Genes

**Figure 2:**
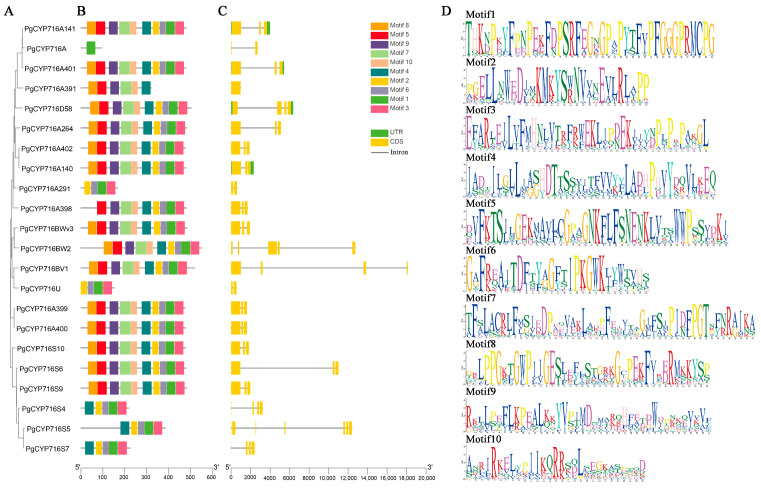
Phylogenetic tree and gene structure analysis of 22 *PgCYP716* genes in *P. grandiflorus*. (**A**) The construction of a phylogenetic tree for the *PgCYP716* gene family. (**B**) The distribution of 10 conserved domains in the PgCYP716. A total of 10 motifs were identified, and each color represents one motif. (**C**) Gene structure analysis of *PgCYP716s*. (**D**) Conserved structural motifs of PgCYP716 proteins. Different colors represented different amino acids.

Plant genes often contain a high frequency of interruptions in the form of introns or exons, offering valuable insights into the evolutionary relationships within gene families. Additionally, the presence of diverse motif compositions can contribute to the functional versatility of genes [32,33]. This study integrated phylogenetic trees, gene structure diagrams, and motif analysis to evaluate the evolutionary relationships and gene structures of the PgCYP716 family. The PgCYP716A had only one motif (motif1). PgCYP716A291 and PgCYP716U had four motifs (motif1, motif2, motif3, and motif6). PgCYP716S4, PgCYP716S5, and PgCYP716S7 in the PgCYP716S subfamily contained five motifs (motif1, motif2, motif3, motif4, and motif6), suggesting that they were evolutionarily more closely related and may have similar functions. PgCYP716A391 and PgCYP716A398 had six and nine motifs, respectively. All the remaining proteins contained 10 motifs (Figure 2B). The gene structure analysis showed that the PgCYP716 family genes had varying numbers of exons, from two to six. Specifically, four genes in this family were found to be devoid of introns, containing only a single exon. (Figure 2C). Meanwhile, among the 10 motifs, some variation in the length of different motifs was observed, suggesting some differences in the functions (Figure 2D).

### 2.4. Analysis of Chromosomal Localization and Duplication of the PgCYP716 Genes

**Figure 3:**
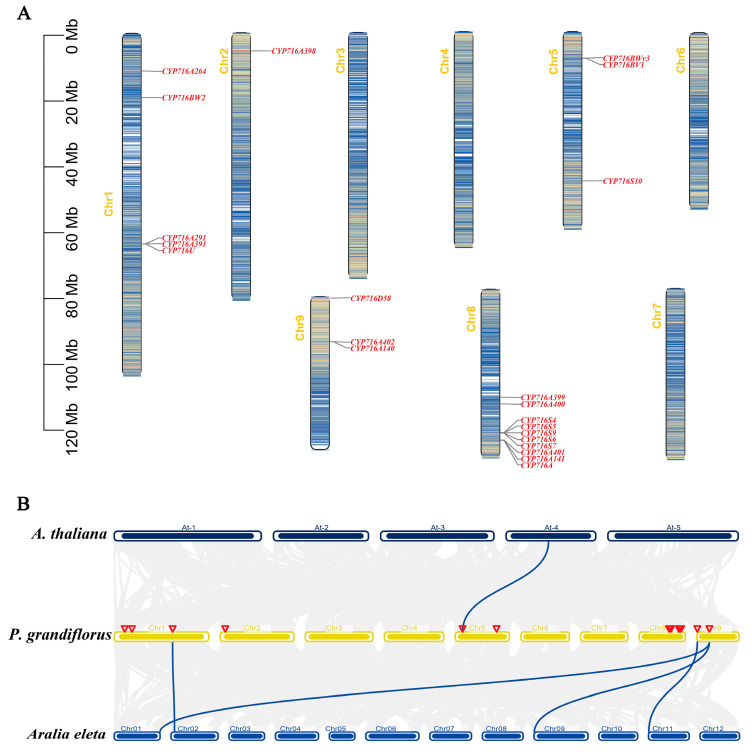
Chromosome localization and collinearity analysis of *PgCYP716* gene family in *P. grandiflorus*. (**A**) Chromosomal distribution of 22 *PgCYP716* genes in *P. grandiflorus*. (**B**) The collinearity analysis of the CYP716 gene family in *P. grandiflorus*, *A. eleta*, and *A. thaliana*. The red triangle represented the chromosomal position of *PgCYP716s*.

By utilizing the genome annotation data of *P. grandiflorus*, our study delved into the precise mapping of *PgCYP716* genes across the chromosomes. The 22 *PgCYP716* genes were mainly distributed on five chromosomes in an uneven manner, with no genes present on chromosomes 3, 4, 6, and 7. Chromosome 8 had the maximum 10 genes, followed by five genes on chromosome 1, three genes each on chromosomes 5 and 9, and one gene on chromosome 2 (Figure 3A).

Gene duplication plays a crucial role in evolutionary dynamics by providing a fundamental mechanism for the expansion and diversification of gene families. This process entails the replication of individual genes within the genome of an organism, resulting in an augmentation of the gene pool through the generation of additional gene copies. The phenomenon of gene duplication underpins the enhancement of genetic diversity, the emergence of novel functions, and the evolution of complex biological systems across various species. The expansion of gene families through duplication events not only contributes to genetic diversity but also provides the raw material for evolutionary innovations and adaptations. To evaluate the evolutionary relationship of the CYP716 genes in various species, collinearity analysis was performed between *P. grandiflorus* and four plants (*P. pseudoginseng*, *P. ginseng*, *A. eleta*, and *A. thaliana*). No collinearity was observed between the CYP716 genes in *P. grandiflorus* and those in the traditional Chinese medicines *P. pseudoginseng* and *P. ginseng*. However, collinearity was observed with those in *A. eleta* and *A. thaliana* (Figure 3B). This indicated that the CYP716 genes in *P. grandiflorus* exhibited relatively distant relationships with *P. pseudoginseng* and *P. ginseng* and closer relationships with model plant *A. thaliana* and a woody plant *A. eleta*.

### 2.5. Analysis of Cis-Acting Element in the Promoter of PgCYP716s

**Figure 4:**
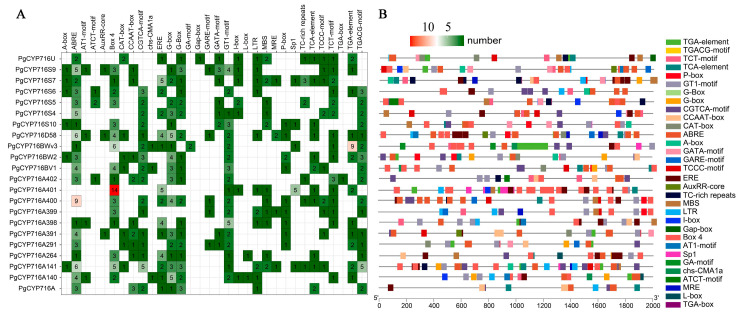
Analysis of cis-acting element in the promoter of *PgCYP716s*. (**A**) The number of different cis-acting elements in the *PgCYP716* gene promoter. (**B**) The positional distribution of different cis-acting elements in the *PgCYP716* gene promoter.

*Cis*-acting elements were mainly involved in the regulation of gene expression. In order to delve deeper into the response of *PgCYP716* genes to challenging conditions, we utilized PlantCARE to identify cis-acting elements from the 2.0 kb 5′ upstream regions of *PgCYP716s*. The *cis*-acting elements present in the PgCYP716 promoters were predominantly associated with the regulation of plant hormones and responses to external environmental stresses (Figure 4). In terms of abiotic stresses, the cis-acting element of the PgCYP716 promoters was observed to be mainly involved in the response to cold stress and drought stress. Additionally, five types of hormone-regulation-related elements were found, including those related to gibberellin, methyl jasmonate, auxin, salicylic acid, and abscisic acid-responsiveness. These findings provide additional evidence that *PgCYP716s* plays a crucial role in the response of *P. grandiflorus* to hormonal signals and environmental stressors.

### 2.6. RNA-seq Analysis of PgCYP716s Gene Expression

**Figure 5:**
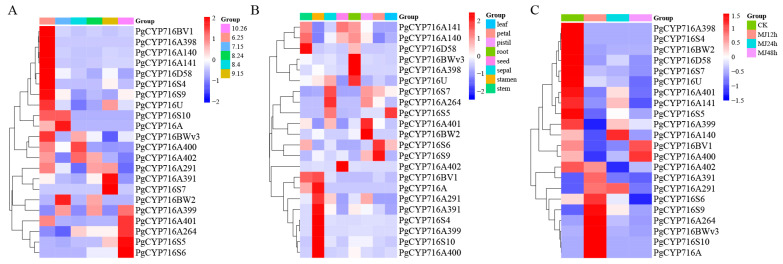
The expression of *PgCYP716s* genes based on RNA-seq. (**A**) The expression of *PgCYP716s* at different levels of saponin accumulation. (**B**) The changes in the expression of *PgCYP716s* in different tissues. (**C**) The changes in the expression of *PgCYP716s* under MeJA treatment.

The content of specialized metabolites changed with the growth cycle of the plant. In order to explore the expression patterns of the *PgCYP716* genes during different periods of saponin accumulation, the expression profiling of *PgCYP716* genes was further investigated based on the transcriptome data from our laboratory. Through analyzing the transcriptome data at different stages of saponin accumulation, the 22 *PgCYP716* genes were roughly divided into two major categories based on similar expressions. One category, represented by genes such as *PgCYP716A140* and *PgCYP716BWv3*, was mainly upregulated in the early stages of saponin accumulation. The other category mainly upregulated in the middle and late stages of saponin accumulation and included genes such as *PgCYP716A391* and *PgCYP716A291* (Figure 5A). This indicated that the *PgCYP716* genes may participate in saponin synthesis at all stages of saponin accumulation.

To evaluate the transcript level of the *PgCYP716* genes in various tissues, the transcriptome data of eight tissues (petal, root, pistil, leaf, flower, seed, stamen, and stem) were downloaded from the SRA database. The results indicated that the 22 genes were expressed in all tissues and could be categorized into three groups based on the expression pattern. The expression of 22 *PgCYP716* genes was the lowest in the roots compared with other tissues. The genes in the first category were mainly upregulated in the root and stem, including *PgCYP716A141* and *PgCYP716BWv3*. The genes in the second category had high degree of expression in the seed, petal, and leaf and included *PgCYP716S6* and *PgCYP716A401*. The genes in the third category possessed a high level of expression in the stamen and included *PgCYP716S10* (Figure 5B). These findings indicated that the *PgCYP716* genes exhibit tissue-specific expression in *P. grandiflorus*.

Analysis of cis-acting elements of the *PgCYP716s* promoter revealed that most of the *PgCYP716* promoters contained a large number of MeJA cis-responsive elements (Figure 5C). To further clarify whether the *PgCYP716* gene responds to MeJA, the transcriptome data downloaded from SRA database were analyzed. The result indicated that most genes could be induced by MeJA, with *PgCYP716A264*, *PgCYP716BWv3*, *PgCYP716A291*, and *PgCYP716A391* getting significantly upregulated at 12–24 h. The expression of *PgCYP716A402* and *PgCYP716BW2* increased at 0 and 12 h and decreased at 24 and 48 h. A class of genes, represented by *PgCYP716A400*, exhibited upregulation mainly at 48 h after induction by MeJA (Figure 5C). These results indicated that the *PgCYP716* genes could respond to MeJA in *P. grandiflorus*.

### 2.7. Cloning and Expression Analysis of the PgCYP716 Genes

**Figure 6:**
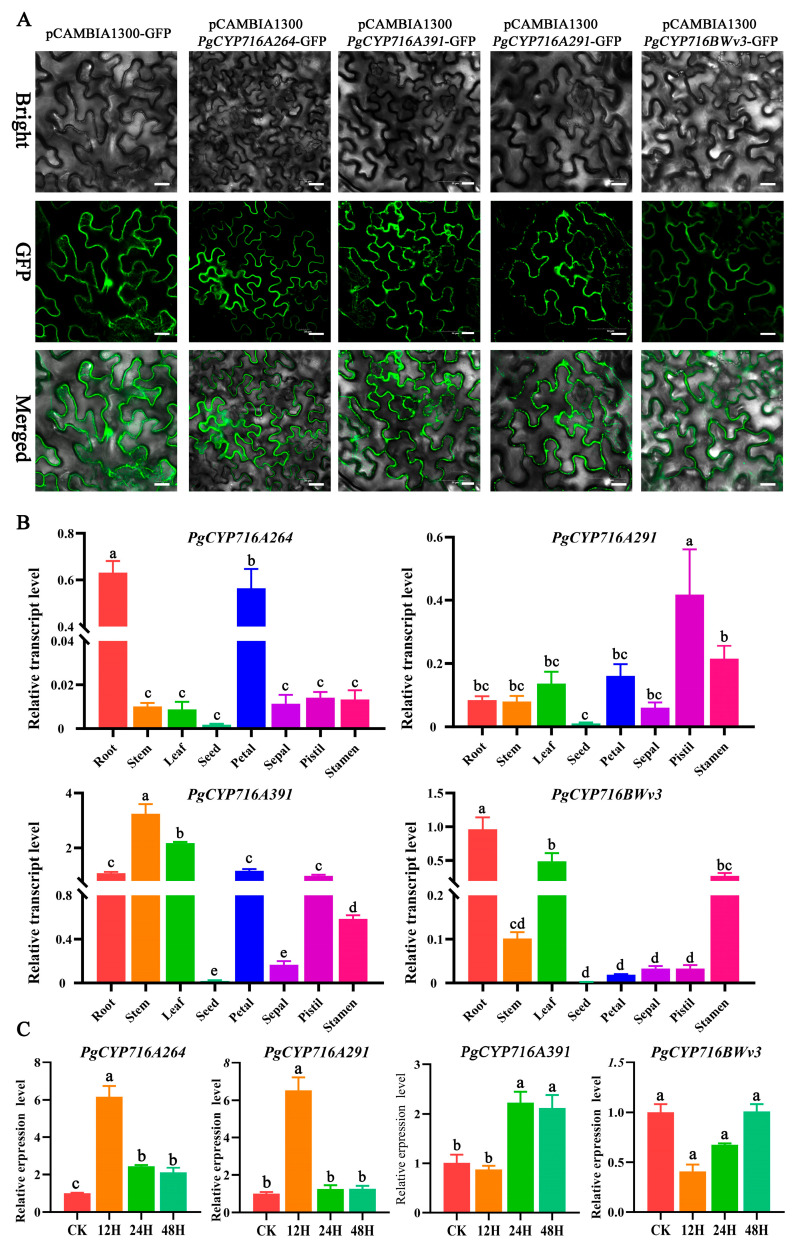
The subcellular localization and expression analysis of *PgCYP716A264*, *PgCYP716A391*, *PgCYP716A291*, and *PgCYP716BWv3*. (**A**) *PgCYP716A264*, *PgCYP716A391*, *PgCYP716A291*, and *PgCYP716BWv3* genes encoded membrane proteins. The top, middle, and bottom represent bright, GFP, and merged fields, respectively. (**B**) Expression levels of the four genes in eight tissues. (**C**) Expression levels of the four genes under MeJA induction. Different letters indicated significant differences (*p <* 0.05). Bar = 20 μm.

RNA sequencing analysis and the sequence similarity of PgCYP716s revealed that the *PgCYP716A264*, *PgCYP716A391*, *PgCYP716A291*, and *PgCYP716BWv3* genes may play an important role in saponin synthesis. *PgCYP716A264*, *PgCYP716A391*, *PgCYP716A291*, and *PgCYP716BWv3* genes were cloned for further research. The subcellular localization experiments indicated that these genes encoded membrane proteins (Figure 6A). Tissue-specific expression data indicated that these genes were expressed at low levels in the seeds compared to other tissues. *PgCYP716A264* and *PgCYP716BWv3* were mainly significantly upregulated in the roots. *PgCYP716A391* was predominantly expressed in the stem, followed by the leaf and stem. The upregulation of *PgCYP716A291* was the highest in the pistil, followed by the stamens and petal (Figure 6B). qRT-PCR analysis of the four genes under MeJA induction revealed that the transcript level of the *PgCYP716BWv3* gene was notably inhibited after MeJA induction. The expression level of *PgCYP716A264* and *PgCYP716A291* reached the highest at 12 h, and *PgCYP716A391* reached the highest at 24 and 48 h (Figure 6C).

### 2.8. The Functional Verification of PgCYP716 Genes in Yeast

**Figure 7:**
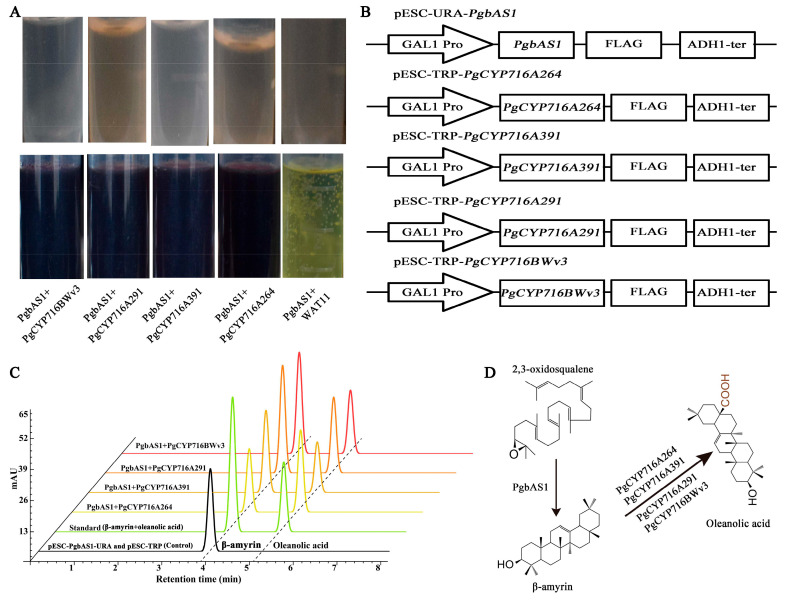
In vivo enzymatic activity assay in yeast. (**A**) Detection of the yeast products using the vanillin-perchloric acid method indicated that the four enzymes could catalyze the production of oleanolic acid from β-amyrin. The top figure shows yeast-induced products, and the bottom figure shows oleanolic acid detected using the vanillin-perchloric acid method. (**B**) Schematic representation of the construction of PgbAS1 and the yeast heterologous expression vectors with four PgCYP716s. (**C**) HPLC analysis of yeast cultures indicated that PgCYP716A264, PgCYP716A391, PgCYP716A291, and PgCYP716BWv3 could produce oleanolic acid. (**D**) A summary of the biochemical reactions catalyzed by four PgCYP716 enzymes.

To verify whether these four PgCYP716 enzymes catalyze the production of oleanolic acid from β-amyrin, the yeast expression vectors of the pESC-*PgbAS1*-URA and pESC-*PgCYP716s*-TRP were constructed and transformed into the WAT11 yeast strain (Figure 7B). The metabolites isolated from yeast culture exhibited a color reaction in the vanillin-perchloric acid assay, indicating the presence of oleanolic acid (Figure 7A). Simultaneously, the metabolites isolated from yeast culture of the four PgCYP716s were concurrently analyzed using HPLC, revealing the presence of oleanolic acid (Figure 7C). These data indicated that all four PgCYP716 enzymes catalyzed the conversion of β-amyrin into oleanolic acid (Figure 7D).

### 2.9. PgCYP716s Overexpression Elevated the Saponin Content

**Figure 8:**
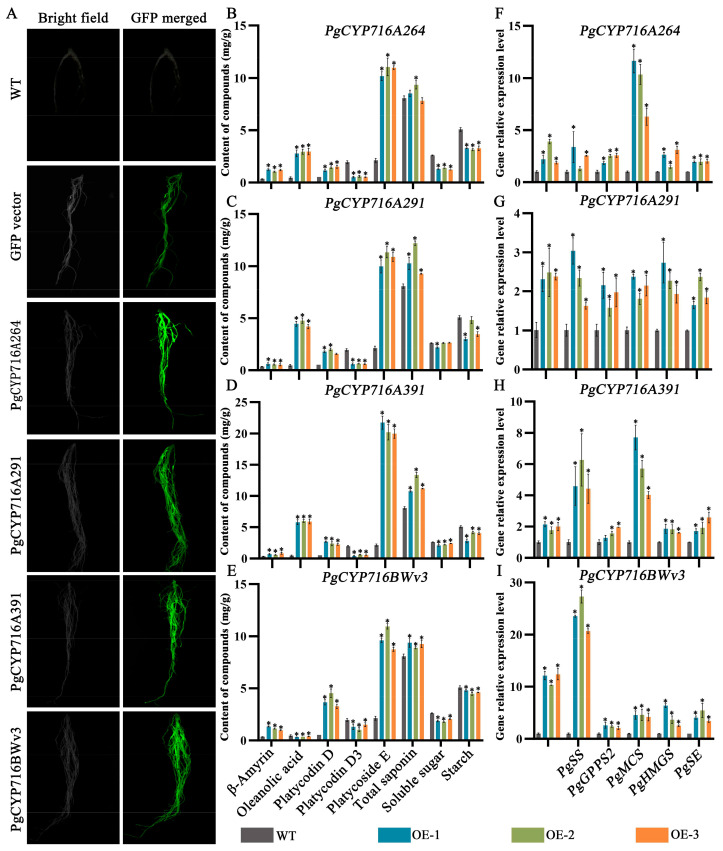
*PgCYP716A264*, *PgCYP716A391*, *PgCYP716A291*, and *PgCYP716BWv3* genes were functionally validated in the hairy roots of *P. grandiflorus*. (**A**) GFP fluorescence was detected in the hairy roots overexpressing *PgCYP716A264*, *PgCYP716A391*, *PgCYP716A291*, and *PgCYP716BWv3* genes. (**B**–**E**) Content of platycoside E, platycodin D3, β-amyrin, oleanolic acid, platycodin D, total saponin, soluble sugar, and starch in the WT and transgenic hairy roots. (**F**–**I**) qRT-PCR analysis of the expression of saponin synthesis pathway genes in overexpressed hairy roots. Asterisks indicated significant differences (*p* < 0.05).

Yeast heterologous expression analysis revealed that all four enzymes catalyzed the conversion of β-amyrin into oleanolic acid. To further confirm the function of these enzymes in *P. grandiflorus* ontogeny, their functions were validated using the hairy root transformation established in our laboratory [34]. The transgenic hairy roots of four *PgCYP716* genes were generated via *Agrobacterium*-mediated transformation (Figure 8A; Supplementary Figure S1). The metabolite content of the transgenic hairy roots was evaluated. The four genes could increase the content of β-amyrin, total saponin, platycoside E, platycodin D3, and platycodin D (Figure 8B–E). Oleanolic acid content in the transgenic hairy roots with overexpressed *PgCYP716A264*, *PgCYP716A391*, and *PgCYP716A291* exhibited an increase, whereas that in the transgenic hairy roots with overexpressed *PgCYP716BWv3* exhibited a decreasing trend (Figure 8E). The macromolecules, namely, soluble sugars and starch, were the key upstream substrates for the synthesis of saponins. In the four transgenic hairy roots, the content of soluble sugars and starch exhibited a decreasing trend compared with the control group. This indicated that in the process of producing downstream specialized metabolites, the upstream macromolecules such as soluble sugars and starch underwent degradation and transformation, leading to reduced content (Figure 8B–E). qRT-PCR was performed to detect the expression levels of functional genes in the saponin synthesis pathway (*PgSS*, *PgGPPS2*, *PgMCS*, *PgHMGS*, and *PgSE*). The results showed that the overexpression of *PgCYP716A264*, *PgCYP716A391*, and *PgCYP716A291* and *PgCYP716BWv3* genes could promote the expression of saponin synthesis pathway genes (Figure 8F–I). These data revealed that four PgCYP716 enzymes participated in catalyzing the production of oleanolic acid from β-amyrin, ultimately contributing to the accumulation of saponin content.

## 3. Discussion

CYP716A is the major subfamily that exhibits catalytic activity in the conversion of β-amyrin to oleanolic acid [35]. Ever since CYP716A12 was first described as a triterpenoid-oxidizing enzyme [17], the CYP716 family has been systematically identified and named in plants such as *Aralia elata* and has been reported to play complex roles in different plants [36]. *P. grandiflorus* contains more than 70 saponin monomers, which mainly belong to oleanane-type saponins [29]. However, systematic identification and nomenclature for the CYP716 family has not been performed. A total of 22 *PgCYP716* genes were identified and classified into seven subfamilies in this study. Multiple sequence alignment results indicated that all 22 genes contained the complete P450 helix K region sequence and heme binding motif (Figure 1A). Through the analysis of the evolutionary tree, the CYP716U, CYP716D, and CYP716BV subfamilies were clustered with the CYP716A subfamily, which catalyzes the synthesis of oleanolic acid from β-amyrin (Figure 1B). It was hypothesized that these three gene families also have the same catalytic function. In addition, the *PgCYP716s* subfamily was clustered with PgCYP716A53v2 from *P. ginseng* (Figure 1B). The function of PgCYP716A53v2 is to catalyze the formation of protopanaxatriol from protopanaxadiol. Therefore, the PgCYP716S subfamily in *P. grandiflorus* may be involved in catalyzing the formation of protopanaxatriol from protopanaxadiol (Figure 1B) [28]. *P. ginseng*, *P. grandiflorus*, and *P. quiquefolium* are traditional Chinese medicinal plants, with saponins as their main active ingredients [37]. No collinearity was observed between the *CYP716* genes in *P. grandiflorus* and those in the traditional Chinese medicines *P. pseudoginseng* and *P. ginseng*; however, collinearity was observed in the model plant A. thaliana and a woody plant *A. eleta* (Figure 3B), presumably due to the different types of saponin compounds.

Various abiotic stresses such as drought, temperature fluctuations, UV radiation, and heavy metals are common challenges in plant growth environments. These environmental factors enhance plant resistance to external stresses by regulating the levels of phytohormones (e.g., methyl jasmonate (MeJA), abscisic acid (ABA), and salicylic acid (SA)) in plants. Phytohormones, as important signaling molecules, play a key role in regulating the synthesis of bioactive compounds in medicinal plants and are also one of the important mechanisms by which external biotic and abiotic stresses promote the accumulation of secondary metabolites in medicinal plants [38]. Analysis of the *cis*-acting elements of the promoter of the *PgCYP716* genes revealed a large number of abiotic stress responses, including cold and drought stress, and hormone response elements, such as MeJA and SA (Figure 4). The analysis of the expression of *PgCYP716s* under MeJA treatment indicated that MeJA could induce the expression of *PgCYP716s* (Figure 5C; Figure 6C). In addition to abiotic stresses, transcription factors play an important role in regulating the biosynthesis of active ingredients in medicinal plants [39]. In *Artemisia annua*, the activation of *AaMYB15*, an R2R3-MYB transcription factor, was triggered by darkness and MeJA treatment. Moreover, *AaMYB15* suppresses the biosynthesis of artemisinin by directly interacting with the promoter of *AaORA* [40]. The promoters of *PgCYP716s* contained a large number of MYB and MYC transcription factor binding sites, suggesting that MYB and MYC transcription factors may affect the accumulation of saponins by regulating the transcription level of *PgCYP716s* (Figure 4).

Five *PgCYP716* genes were identified and functionally verified in *P. grandiflorus*; they catalyze the synthesis of oleanolic acid from β-amyrin (Figure 1B). For more comprehensive understanding of the functions of the remaining genes in this family, we cloned the remaining 17 genes of this family, successfully isolating 11 genes. We speculated that the genomic heterozygosity of *P. grandiflorus* is too high or repetitive sequences may be folded during the assembly process, leading to assembly errors and affecting the gene cloning [35,41,42]. Based on the sequence similarity of PgCYP716s and the gene expression changes under MeJA treatment and different saponin accumulation periods, the function of *PgCYP716A264*, *PgCYP716A391*, *PgCYP716A291*, and *PgCYP716BWv3* genes were further studied. Similar to the majority of CYP450 proteins, subcellular localization analysis revealed that the four PgCYP716 genes in *P. grandiflorus* encoded membrane proteins (Figure 6A) [36,43]. Yeast heterologous expression revealed that PgCYP716A264, PgCYP716A391, PgCYP716A291, and PgCYP716BWv3 oxidize β-amyrin to produce oleanolic acid (Figure 7). This indicated that the CYP716BWv subfamily may also play an important role in catalyzing the production of oleanolic acid from β-amyrin [44]. Through hairy root transformation, transgenic and positive hairy roots of four genes were successfully obtained (Figure 8A; Supplementary Figure S1). Overexpression in hairy roots showed a notable enhancement in β-amyrin and total saponin levels in comparison to the wild-type (WT). Gene expression analysis further confirmed that the genes involved in the saponin biosynthesis pathway were up-regulated in the overexpressed hairy roots (Figure 8B–I). However, overexpression of the *PgCYP716BWv3* gene resulted in decreased oleanolic acid content (Figure 8E). This suggested that PgCYP716BWv3 has a continuous catalytic function, catalyzing the formation of other downstream substances after producing oleanolic acid and leading to its reduced content [23]. Platycodin D, platycodin D3, and platycoside E are the main saponin monomers in the *P. grandiflorus* root [1]. Though the contents of platycodin D and platycoside E increased in overexpressing hairy roots compared with WT, the content of platycodin D3 decreased (Figure 8B–E). This indicated that enzymes involved in later modifications (e.g., PgCYP450 and PgUGT) may contribute to the differences in the content of different saponin monomers.

## 4. Materials and Methods

### 4.1. Identification of the PgCYP716 Genes

The genome data of *P. grandiflorus*, CYP450 protein sequence of *A. thaliana* and annotation file of CYP450 domain (PF00067) were downloaded from the NGDC (https://ngdc.cncb.ac.cn/gwh, accessed on 15 May 2024), TAIR database (https://www.arabidopsis.org/, accessed on 15 May 2024), and Pfam database (http://pfam-legacy.xfam.org/, accessed on 15 May 2024), respectively [33,41]. Initially, the BioEdit was used to screen the genome of *P. grandiflorus* through BLASP search, using the AtCYP450 protein sequence as the query with a threshold of *E*-value ≤ 10^−5^ to acquire the initial set of candidate genes [45]. Subsequently, the HMM (PF00067) was applied to build the HMM profile against the *P. grandiflorus* protein dataset (*E*-value ≤ 10^−10^ and bits ≥ 85) to obtain a secondary set of candidate genes. After merging and removing duplicate sequences from both gene sets, the resulting amino acid sequences of the CYP450 family of *P. grandiflorus* were forwarded to the Cytochrome P450 Homepage (https://drnelson.uthsc.edu/, accessed on 5 May 2024) for nomenclature. Finally, the number of *PgCYP716* genes was determined. Additionally, the features of the PgCYP716 sequences were determined using the ExPasy website (https://web.expasy.org/protparam/, accessed on 15 May 2024).

### 4.2. Phylogenetic Analysis and Classification

The MUSCLE software (v 3.8.31) was employed to align the amino acid sequences from *P. grandiflorus* (Pg), *Vitis vinifera* (Vv), *Maesa lanceolata* (Ml), *Chenopodium quinoa* (Cq), *Barbarea vulgaris* (Bv), *Glycyrrhiza uralensis* (Gu), *Catharanthus roseus* (Cr), and *Bupleurum falcatum* (Bf). Subsequently, the phylogenetic tree was constructed via MEGA 11 (https://www.megasoftware.net/, accessed on 15 May 2024) with the Maximum likelihood method [46]. To enhance the stability of the tree, the Bootstrap parameter was set to 1000. Subsequently, the ggtree package was used to visualize the phylogenetic tree [47].

### 4.3. Analysis of the Gene Structure and Conserved Protein Motifs of PgCYP716 Genes

The MEME online tool (http://meme-suite.org/meme/tools/meme, accessed on 5 May 2024) was utilized to predict the motif positional distribution and quantity within the PgCYP716 protein sequences. The analysis was conducted with specified parameters, setting a maximum of 10 deviations and an optimal motif width ranging from six to 200 amino acid residues. TBtools (v 2.088) was used to analyze the intron–exon structure of the *PgCYP716* genes. Finally, the evolutionary tree, gene structure, and conserved domains of CYP716 were visualized using TBtools [48].

### 4.4. Analysis of Cis-Acting Elements in the Promotors of PgCYP716 Genes

To further explore the regulatory elements of the PgCYP716 gene family, the sequence 2000 bp upstream of the translational start codon of *PgCYP716* genes was extracted using the Seqkit (v0.11.0), bedtools (v2.30.0), and samtools packages (v1.6) as described in previous studies [49,50,51]. Subsequently, PlantCARE (http://www.plantcare.co.uk/, accessed on 15 May 2024) was used to predict cis-acting elements. Finally, cis-acting element positions and numbers were visualized using TBtools, tidyverse, ggplot2, and RColorBrewer packages [48,52].

### 4.5. Chromosome Localization and Gene Collinearity

The chromosome position of the *PgCYP716* genes were extracted based on the annotation file of the *P. grandiflorus* genome. The genomic data of *A. thaliana*, *P. notoginseng*, *P. ginseng*, and *Aralia elata* were downloaded from the Tair database (https://www.arabidopsis.org/, accessed on 15 May 2024) and the TCM Plant Genome Database (http://cbcb.cdutcm.edu.cn/TCMPG/, accessed on 15 May 2024). TBtools was used for collinearity analysis and visualization [48].

### 4.6. Expression Pattern of PgCYP716s Based on RNA-seq

The transcriptome data of eight different tissues (SRR8712510–SRR8712517) and the root treated with MeJA (SRR8712518–SRR8712529) were downloaded from the SRA database. HISAT2 (v2.2.1) and featureCounts (v2.0.1) were used to analyze the transcriptome data and obtain gene expression matrices [53,54]. Additionally, based on the existing transcriptome data of root saponin accumulation from June to October in our laboratory, the expression level of the *PgCYP716* genes in different periods of saponin accumulation was analyzed. Finally, heatmap package (v1.0.12) was used to construct the diagrams [55].

### 4.7. Treatment of P. grandiflorus Roots with MeJA

In this experiment, 30-day-old seedlings of *P. grandiflorus* were cultivated on 1/2 MS medium containing 30 g/L sucrose and 5 g/L agar (pH 5.8). The seedlings were further treated with 100 μM MeJA dissolved in dimethyl sulfoxide within a growth environment of 16 h light followed by 8 h of darkness, maintained at a temperature of 25 °C. The samples treated with MeJA were collected at 0, 12, 24, and 48 h.

### 4.8. Cloning of Full-Length of PgCYP716s and PgbAS1

Total RNA from *P. grandiflorus* was extracted using Trizol method and the RNA was converted into cDNA using reverse transcription kit (Takara, Beijing, China). The sequences of four *PgCYP716* genes and the *PgbAS1* sequence (Accession number: KY412556.1) were obtained from the genome database of *P. grandiflorus* and the NCBI database, respectively. The open reading frames of *PgbAS1* and *PgCYP716s* were amplified from the *P. grandiflorus* cDNA via PCR using KeyPo DNA polymerase (Vazyme Biotech Co., Ltd., Nanjing, China). Additionally, the DNA sequences were inserted into the pMD19-T vector (Takara, Beijing, China) for cloning, followed by sequencing to verify their accuracy and integrity. Primers used to amplify both *PgCYP716s* and *PgbAS1* genes were designed using Primer premier 5. The primers used in this step are all listed in Supplementary Table S1.

### 4.9. Quantitative Real-Time PCR

Plant RNA was acquired from eight different tissues (roots, stems, leaves, petals, stamens, pistils, petals, and seeds) and the root tissue treated with 100 μmol MeJA for 0, 12, 24, and 48 h. qRT-PCR was conducted using the BIO-Rad CFX96 instrument. The *18S rRNA* gene was used as the internal reference gene in this study [1]. All primers are listed in Supplementary Table S2.

### 4.10. Yeast Heterologous Expression

The full-length 2,3-oxidosqualene cyclase *PgbAS1* gene was inserted into pESC-URA vector, and the full-length of *PgCYP716* genes were inserted into the pESC-TRP vector. The engineered yeast strain Saccharomyces cerevisiae WAT11, which has been modified to incorporate the P450 reductase from *A. thaliana*, serves as an optimal heterologous host for the expression of P450 enzymes. This strain is equipped with the mevalonate (MVA) metabolic pathway, enabling it to synthesize the triterpenoid precursor 2,3-oxidosqualene [56]. To validate the function of the *PgCYP716s*, the pESC-*PgbAS1*-URA vector was used to transform WAT11, after which the cyclization of 2, 3-oxidosqualene was catalyzed to generate β-amyrin [57]. Further, the pESC-*PgCYP716s*-TRP vector was used to transform the WAT11 containing pESC-*PgbAS1*-URA as described previously [10]. pESC-*PgbAS1*-URA and pESC-TRP (the empty vector) were also co-transformed into WAT11 as the control. The transformed yeast was inoculated in 5 mL SD/-TRP-URA liquid medium and grown till the OD_600_ reached 1.5. Further, the yeast cells were harvested by centrifugation at 1000 rpm for 1 min, washed two times using sterile water, resuspended in 100 mL SC/-TRP-URA liquid medium, which added 2 g galactose, and cultured for 3 days in the dark at 28 °C to induce metabolite biosynthesis in yeast. Further, we use a mixture of acetone and ethyl acetate (1:1 *v*/*v*) to extract yeast metabolites as described previously [56]. The liquid was evaporated using a low-temperature concentration method, and the remaining residue was dissolved in 1.5 mL methanol for the next step of analysis. The metabolites isolated from yeast culture were detected using the vanillin-perchloric acid method and high-performance liquid chromatography (HPLC) [58,59]

### 4.11. Subcellular Localization

The full coding sequences of *PgCYP716* genes lacking a stop codon were inserted into the pCAMBIA1300-GFP vector at the *Bam*HI and *Kpn*I sites. Subsequently, the sequences were aligned with the genome sequences, and the correctly aligned sequences of pCAMBIA1300-*PgCYP716s*-GFP vector were used to transform *Agrobacterium tumefaciens* GV3101 by referring to the instructions from GV3101 Chemically Competent Cell (Weidibio, Shanghai, China). Cell suspensions with pCAMBIA1300-*PgCYP716s*-GFP vectors were transiently injected into 4-week-old tobacco leaves. After 2 days of incubation in the dark at 23 °C, the subcellular localization of the PgCYP716s was visualized and photographed using a laser-scanning confocal microscope at 488 nm (FV3000, Olympus, Tokyo, Japan).

### 4.12. Functional Verification of PgCYP716 Genes Using Hairy Root Transformation

The *PgCYP716* genes were integrated into the pCAMBIA1300-sGFP vector for overexpression and then introduced into the *A. tumefaciens* strain K599. After selecting single colonies, they were cultured in 100 mL LB medium, which contained 50 mg/L kanamycin and 200 μmol/L acetosyringone (AS), until reaching an OD_600_ of about 0.8. Subsequently, the cells were collected by centrifugation at 4000 rpm for 20 min, resuspended in infiltration solution (200 μmol/L AS, 10 mmol/L 2-Morpholinoethanesulphonic acid (MES), and 10 mmol/L MgCl_2_). This cell suspension was used to infect 30-day-old seedlings without roots using the vacuum infiltration method as described in a previous study [34,60,61]. Further, the seedlings were cultivated in sterilized soil. Positive hairy roots were identified after 60 days and measured for the target gene expression and saponin content.

### 4.13. Statistical Analysis

All experiments were conducted with three biological replicates. Results are reported as mean ± standard error (SE). GraphPad Prism 8.0 (San Diego, CA, USA), Origin (Pro) (OriginLab Corporation, Northampton, MA, USA) and R were used to plot graphs. Asterisks denoted statistical significance using Student’s *t*-test or one-way analysis of variance (ANOVA). Different letters indicate significant differences (*p*  <  0.05).

## 5. Conclusions

A total of 22 *PgCYP716* genes from seven subfamilies were identified in this study. The PgCYP716 gene family was comprehensively analyzed through phylogenetic, collinearity, cis-acting element, and gene expression analyses in different tissues under MeJA treatment and at various developmental stages. Four genes, namely, *PgCYP716A264*, *PgCYP716A391*, *PgCYP716A291*, and *PgCYP716BWv3*, were finally cloned. Subcellular localization, yeast heterologous expression, and overexpression in hairy roots revealed that all four PgCYP716 enzymes were membrane proteins. All of them could catalyze the formation of oleanolic acid from β-amyrin, thereby increasing the content of saponin. This study laid the foundation for further understanding of the role of *PgCYP716* genes in saponin synthesis, providing suitable candidate genes for breeding varieties of *P. grandiflorus* that produce high levels of saponin.

## Data Availability

The original contributions presented in the study are included in the article/Supplementary Materials, further inquiries can be directed to the corresponding author/s.

## Supplementary Materials

The following supporting information can be downloaded at: https://www.mdpi.com/article/10.3390/plants13141946/s1, Figure S1: Detection of transgenic hairy roots using agarose gel electrophoresis; Table S1: Prediction of physicochemical properties of PgCYP716 family proteins; Table S2: Primer design of qRT-PCR and vector construction.
