# Supplementary material for: Genome-Wide Identification of the CYP716 Gene Family in Platycodon grandiflorus (Jacq.) A. DC. and Its Role in the Regulation of Triterpenoid Saponin Biosynthesis
Source: Plants (Basel). 2024 Jul 16;13(14):1946. doi: 10.3390/plants13141946 (PMC11281222; doi:10.3390/plants13141946)
Supplement: Supplementary file 1 [file plants-13-01946-s001.zip › Supplementary Figure S1.pdf]

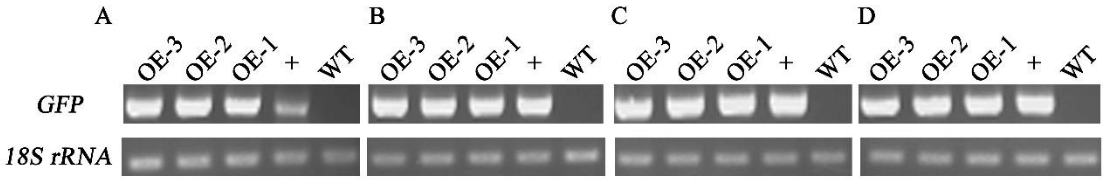

Figure S1. Detection of transgenic hairy roots using agarose gel electrophoresis (A)–(B) Agarose gel electrophoresis detected the GFP gene in the overexpressing transgenic hairy roots of *PgCYP716A264*, *PgCYP716A391*, *PgCYP716A291*, and *PgCYP716BWv3*.
